# An in silico study of tymovirus virocells

**DOI:** 10.1099/jgv.0.002329

**Published:** 2026-10-01

**Authors:** A.J. Gibbs, M.J Gibbs, H.M Gibbs, M. Hajizadeh

**Affiliations:** 1 The Emeritus Faculty, Australian National University, Canberra, ACT, Australia; 2 Independent Researcher, Canberra, ACT, Australia; 3 Plant Protection Department, Faculty of Agriculture, University of Kurdistan, Sanandaj, Iran

**Keywords:** AlphaFold3 modelling, intrinsically disordered proteins, movement protein, membrane targeting, protein polymers, tymoviruses

## Abstract

We used computational tools to study the tymoviral proteome. Past gene sequence comparisons had shown that tymovirus genomes encode five proteins already identified in other viruses; however, our AlphaFold3 modelling found, in addition to their five *α*-helical and *β*-sheet structures, five regions of ‘intrinsically disordered protein’ (IDP). Notably, one of these IDPs is the movement protein (MP), distinguishing it from the structured MPs of other plant viruses. The MP is the largest IDP having some features of structured proteins including genus-specific conserved motifs. All tymovirid methyltransferases (Mtases) have a novel structure that includes a membrane-targeting domain (MTD) at their C-termini. Modelling showed that polymers of 3 or 11 copies of the Mtase:MTD are favoured; the former form complexes with the viral RNA-dependent RNA polymerases, whereas the latter form short rounded cylinders with an equatorial groove. The cylinders are similar in size to the necks of vesicles previously found in the outer chloroplast membranes of all tymovirus-infected plants. The DisLipPred program found that the outer groove of the polymeric cylinders was probably lipophilic and so able to associate stably with plasma membranes. The vesicles are probably the ‘replication organelles’ of tymoviruses and the cylinders possibly the ‘replication organelle organizers’. Modelling also indicated that the tymovirus helicase may function as a tetramer and that the coat protein may form trimer intermediates for assembling the virions. Our study has revealed some of the complexity and three-dimensionality of the viral components of tymovirus virocells.

## Data Availability

The primary gene sequence data from sources are listed in the text.

## Introduction

Markham and Smith [1, 2] first isolated turnip yellow mosaic virus (TYMV) from turnips in south-east England and found that it was sap- and beetle-transmitted and that the ‘virus’ was a nucleoprotein and could be purified and crystallized. Early electron microscope studies of negatively stained TYMV particles [3, 4] showed them to be icosahedral with 32 knobs, and the detailed structure of these virions was later revealed by X-ray crystallography [5–7]. Ultrastructural studies [8–12] showed that 3 days after inoculation with TYMV, the cells in Chinese cabbage leaves have rounded and aggregated chloroplasts with small vesicles attached to the inner surfaces of their outer membranes (Fig. 1). These vesicles were also found in the chloroplasts of the midribs, in leucoplasts of roots and chromoplasts of petals, and they were found in all the infected host plant species of TYMV and other tymoviruses. Their discovery led Matthews [12] to state that ‘TYMV appears to be a virus that has adapted to parasitize primarily one organelle - the chloroplast - rather than the cell as a whole’. Similar vesicles, now called ‘replication organelles’ (ROs), are a feature of cells infected with plant and animal viruses with single-stranded positive-sense RNA genomes [13–17].

**Fig. 1.**
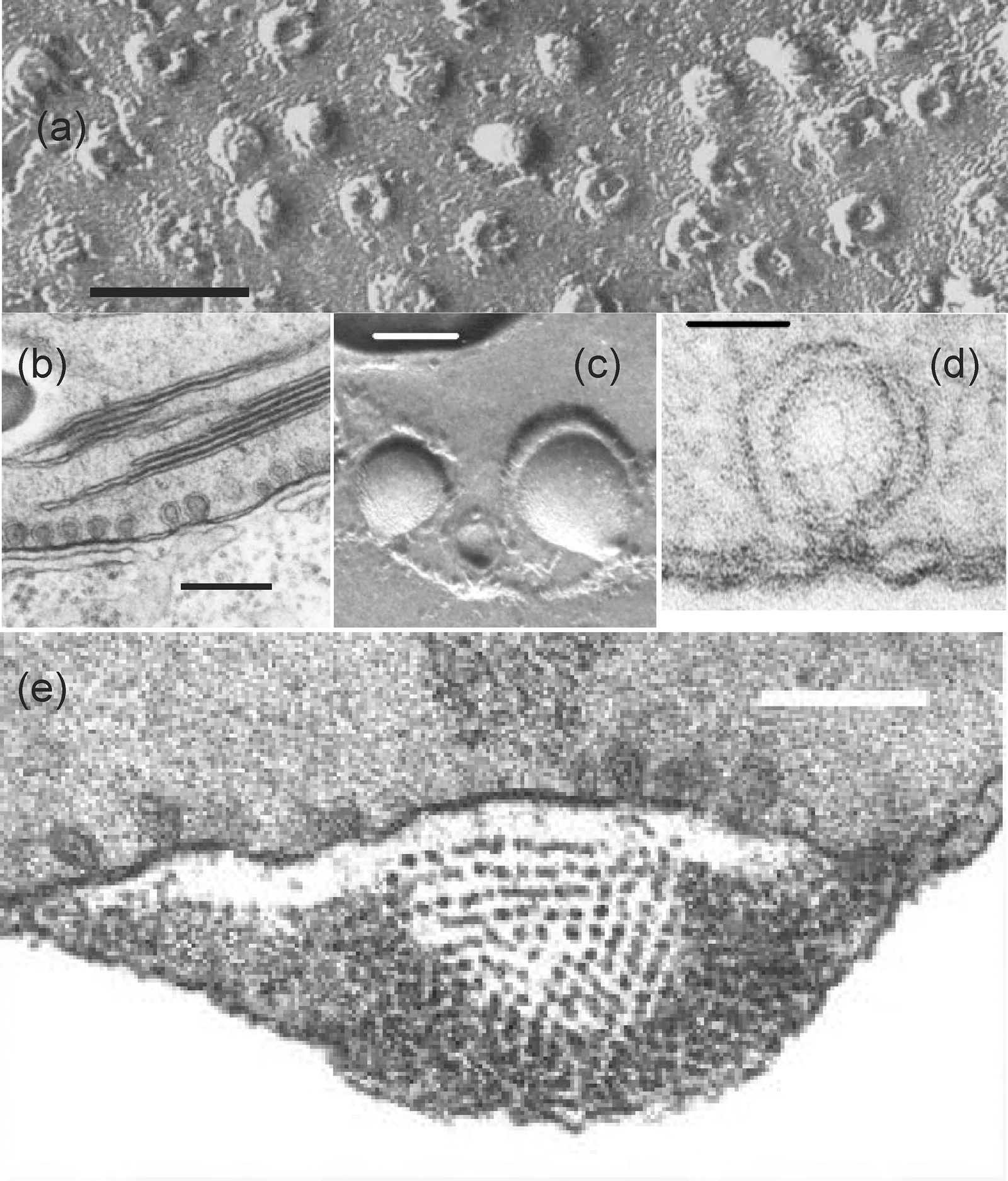
Electron micrographs of TYMV-infected plant tissue. (**a**) Freeze-fractured outer surface of the inner lipid layer of chloroplasts (bar 100 nm). (**b**) Section of an infected chloroplast (bar 250 nm). (**c**) Freeze-fractured vesicles (bar 100 nm). (**d**) Section of a vesicle (bar 50 nm). (e) Section of an infected plasmolysed chloroplast (bar 250 nm). Photos reproduced from Descriptions of Plant Viruses No. 280 with permission of the Association of Applied Biologists.

Biochemical studies have shown that the genome of TYMV is a positive-sense RNA of 5,900–6,500 nts [18], and two of its ORFs and encoded proteins [19] are related to those found in a wide range of viruses and appropriately named ‘hallmark genes’ [20]. However, its third ORF encodes the movement protein (MP) and is unlike the MPs of other viruses; it initiates seven nts closer to the 5′ end of the genome than the main ORF and overlaps it. The MP is of 69 kDa [18] (Fig. 2a) and is required for intercellular spread of the virus [21], but not for replicating in isolated cells [22], and it also inhibits post-transcriptional gene silencing [23]. The MP is also of interest as Gibbs *et al*. [24, 25] reported that the MP ORFs of Andean potato latent virus (APLV) and TYMV are positively selected, whereas all the others are under strong negative selection, suggesting the MPs may be the tymovirus avirulence genes [26].

**Fig. 2.**
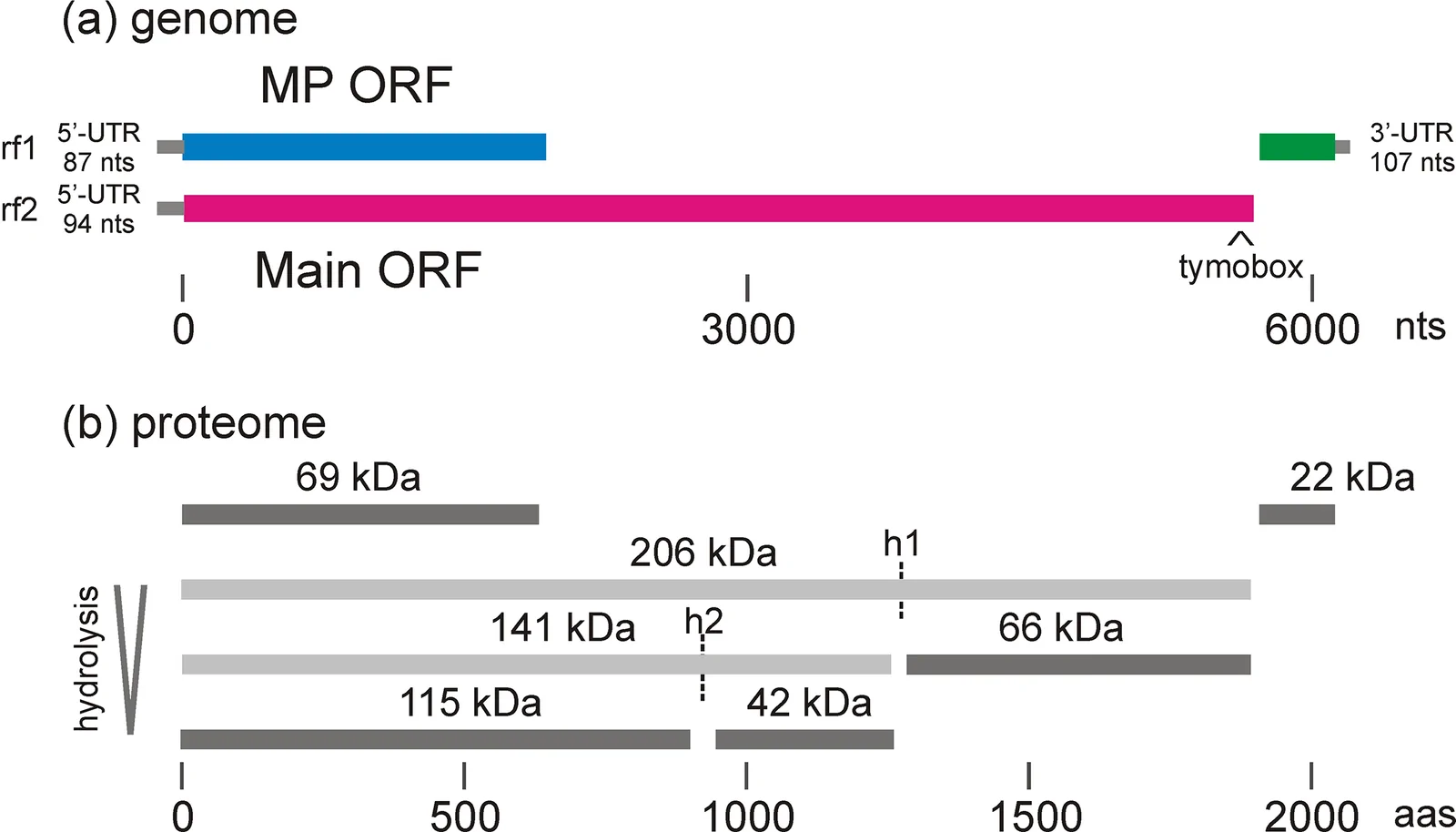
(**a**) TYMV genome of 6000 nts with MP ORF and main ORF reading frames. Proteome diagram shows the 206 kDa protein cleaving at hydrolysis sites h1 and h2 into 141 kDa, 115 kDa, 66 kDa and 42 kDa fragments.

The main features of the TYMV genome and proteome are shown in Fig. 2. The longest tymovirus ORF produces a protein of 206 kDa (Fig. 2b) with, near its 3′-terminus, a sequence feature named the ‘tymobox’ [27, 28] that promotes transcription from the 5′-terminal region of the complete negative genome to produce subgenomic mRNAs of the coat protein (CP) of ca. 22 kDa [28]. The 206 kDa protein is autolysed at the h1 site (Fig. 2b) by the peptidase (Pep) protein of the 141 kDa protein into an N-terminal 141 kDa part and a 66 kDa C-terminal part [21, 29]. The 141 kDa portion is further hydrolysed at the h2 site (Fig. 2b) [30] by the same viral Pep into an N-terminal protein of 115 kDa and a C-terminal part of 42 kDa [30]. Thus, the 206 kDa protein hydrolyses into three parts: a 115 kDa protein that has methyltransferase (Mtase) and Pep regions, the 42 kDa protein that has the sequence of a viral helicase (Hel) [31] and the 66 kDa protein which is an RNA-dependent RNA polymerase (RdRp).

Our study of the tymovirus proteome reported here aimed to determine whether recently developed computational methods, such as AlphaFold3 (AL3), could provide information on the shape and functioning of the MPs and hallmark proteins of tymoviruses. Modelling of the structures suggested that some of the proteins polymerize and become involved in the ROs of tymovirus. Forterre [32] defined viruses as micro-organisms with a two-part life cycle, alternating between metabolically inert virions that disperse the virus and metabolically active ‘virocells’ (i.e. infected host cells) that produce ROs and virions. Thus, in summary, we have used computational methods to better understand the structure and function of proteins contributed by tymoviruses to their virocells

## Methods

### Sequences

The alignment of 107 complete genomic sequences of plant-infecting tymoviruses assembled by Gibbs *et al*. [25] was used for the studies reported here. The sequences included 21 sequences of TYMV and 18 of APLV. For some comparisons, we minimally used the proteins of erysimum latent virus (ELV-Cz), which is the tymovirus phylogenetically most distant from TYMV, also dulcamara mottle virus (DMoV), kennedya yellow mosaic virus (KYMV), ononis yellow mosaic virus (OYMV) and APLV, as these are phylogenetically distant from TYMV and/or have different natural hosts. We believe these comparisons provide a ‘biological variation test’ of the protein modelling predictions, indicating they are robust.

### Analysis

Sequences were edited using BioEdit [33], their encoded aa sequences were aligned using MAFFT [34] or mega11 [35] with default parameters and the nt sequences were aligned using the online Pal2Nal server [36] and the aa alignment from MAFFT as a guide. The most appropriate models for sequence comparisons were calculated using mega11, and the best were found to be GTR+G+I [37] for the hallmark nt sequences, LG+G+I [38] for most aa sequences, but JTT+G+I or WAG+G+I for the IDP sequences.

### Databases

EMBL’s InterPro database (https://www.ebi.ac.uk/interpro/), now including Pfam, was used to search for known aa motifs. Meme, Streme and Xstreme (Meme Suite; https://meme-suite.org/meme/ [39]) were used to search for motifs. HHBlits searches were done at https://toolkit.tuebingen.mpg.de/tools/hhblits. A FoldSeek search (https://search.foldseek.com/search) was made of the Big Fantastic Virus Database [40]; it contains virus protein structures predicted by ColabFold 1.5.5 from the UniRef30 and other databases.

### Molecular modelling

AlphaFold 3 (AL3) [41] online was used for modelling and docking tests of aa sequences. The ‘predicted template modelling (pTM) score’ and the ‘interface pTM (ipTM) score’ were used to assess the accuracy of models [42, 43] and also to assess the likelihood that multiple copies of individual TYMV proteins will form polymers or the likelihood that different TYMV proteins will associate to form oligomers; this seemed a logical use of these measures; however, it has subsequently been shown [42] to ‘accurately predict oligomeric states and structures for a range of soluble and membrane proteins’. pTM and ipTM scores >0.5 indicate that the structure is probably correct. Models calculated by AL3 and stored as ‘cif’ files were examined using Microsoft Visual Studio (VS) Code, Pymol (Schrödinger) and Mol*Viewer online (https://molstar.org/viewer/), and some were converted to Protein Data Bank (PDB) format for searching the Nomberg *et al*. [44] database of predicted protein structures from >4,400 eukaryotic viral species. Mol*Viewer online was also used to estimate the sizes of AL3 models. In-house computer applications were programmed in Python 3.10+. The likely binding propensities of different parts of the TYMV proteins were determined using DisoLipPred [45] and DisoRDPbind [46].

The canonical sizes of cell, chloroplast and plasmolemma membranes and plasmodesmata were obtained from various sources, and most are not referenced individually.

## Results

### Protein structures

We predicted the structure of the proteins of TYMV using AL3 [41] (Fig. 3a, b). It was known from past sequence comparisons [19] that tymovirus genomes encoded five hallmark proteins already identified in other viruses. AL3 modelling found that, in addition to the core domains of the five named hallmark proteins, indicated by structures of *α*-helical and *β*-sheet regions, there were also five major and some smaller regions of ‘intrinsically disordered protein’ (IDP) between some of them. The positions of these regions for the TYMV genome are listed in Table 1.

**Table 1.** Regions of the hallmark genes and IDPs of the TYMV genome

	Size (kDa)	Name	N-terminus	C-terminus	Length (aas)
Overlapping ORF	69	MP	MSNGL-	-APDTD	628
Main ORF	115	Mtase	MAFQL-	-DNLQT	355
		Membrane-targeting domain (MTD) including IDP1	FALLN-	-RTPLS	211
		IDP2	IPRTT-	-PSSQL	165
		Pep	LPAPL-	-PSAKG	154
	42	Hel	HPSDP-	-KLNGA	374
	66	IDP3	TPSAS-	-DTHFL	48
		RdRp	PPSRL-	-LHYVQ	537
	22	IDP4	MEIDK-	-AVPGP	26
		CP	SPLTI-	-TDTST	163

**Fig. 3.**
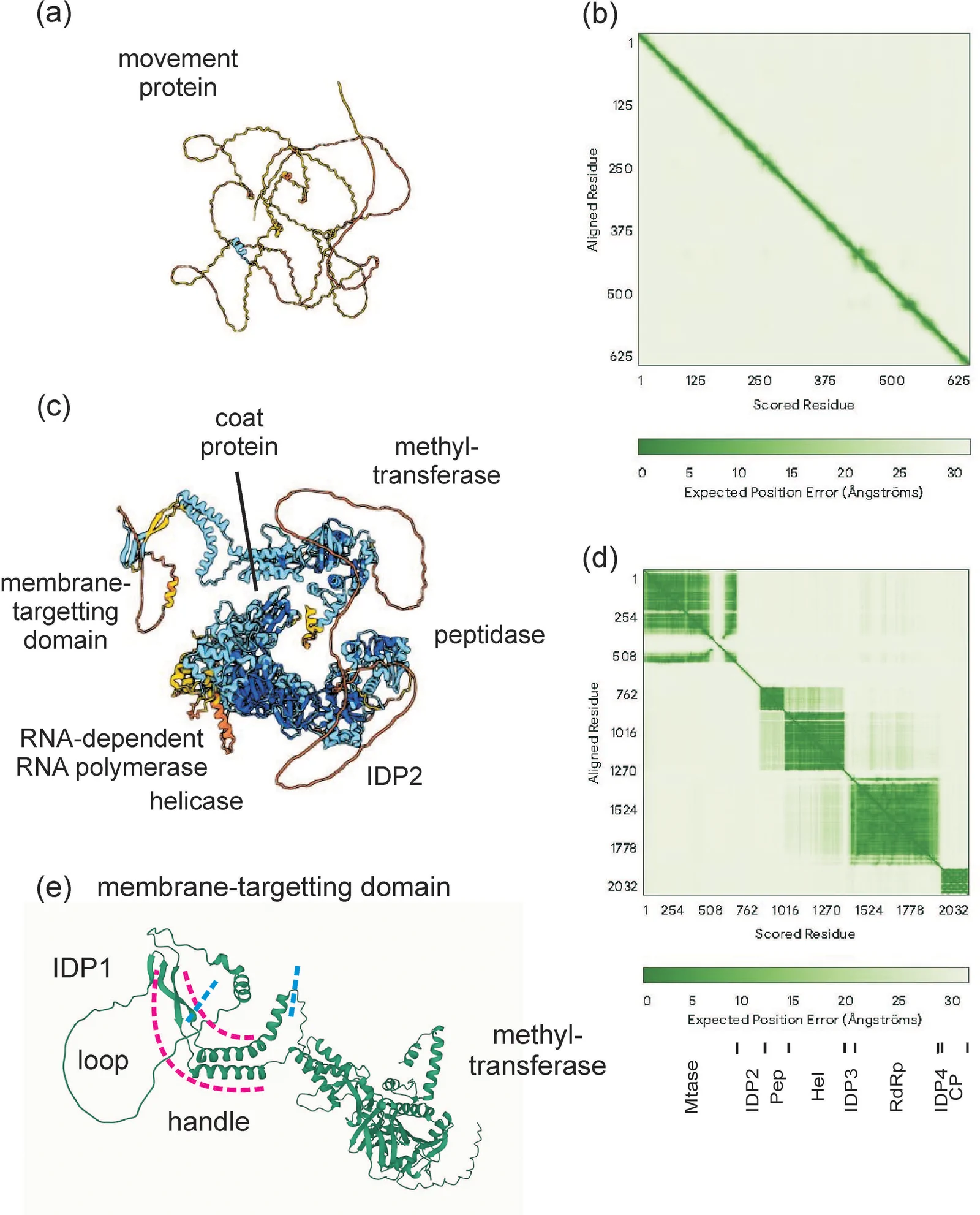
AL3 model showing ribbon diagrams of TYMV 69 kDa and 206 kDa proteins with EPE plots showing diagonal-only shading for IDPs versus block off-diagonal patterns for structured domains including Mtase, helicase, RdRp and peptidase.TYMV’s

Regions with similar sequences and structures were found in Andean potato mild mottle virus (APMMV), APLV, DMoV, ELV-Cz strain, KYMV and OYMV. Their MPs vary most in length, from the shortest which are of belladonna mottle virus and DMoV (576 codons each, 62.0 kDa) to the longest which is that of KYMV (758 codons; 82.4 kDa). IDP1s vary from 37 to 95 codons in length. IDP2s separate the Mtase protein from the Pep protein and are from 74 to 203 codons in length. There is no significant IDP between the Pep and Hel proteins; but this is the h2 proteolytic cleavage site (47). IDP3s are at the N-terminus of their RdRps and are of 47 to 65 codons. The shortest IDP is IDP4 at the N-terminus of the CP and is incorporated into the virion, varying from 26 to 38 codons. When the lengths of each of the IDPs of 107 different viruses were plotted against one another pairwise, most fell into single clusters, but a few were consistent outliers: ELV (NC_001977) had a short IDP1, but a long IDP2, -3 and -4, KYMV had long IDPs -2 and -3 and UllucusTV3 had a long IDP2.

### 69 kDa – the MP

Modelling showed all the tymovirus MPs to be mostly intrinsically disordered (IDPs) (Fig. 3) and unlike those previously reported for other plant virus MPs [47, 48]. Mahillon *et al*. [49] reported that the N-terminal part of the 702 aa MP of alcea yellow mosaic tymovirus has several conserved blocks of positively charged aas, but an HHBlits search found no matches for them. We found, however, that when MP sequences of 107 tymovirus [25] are aligned, either as the encoding nts or as the encoded aas, their N-terminal halves align with only one short region needing gaps for alignment (7% gaps in total), whereas the C-terminal halves require many gaps to align (53% gaps). Thus, the N-terminal region of MPs is as conserved as a hallmark protein, whereas their C-terminal regions are as variable as IDPs. No similarities with structures in the Big Fantastic Virus Database [40] were found. The 107 tymovirus MPs had 2 short regions with 100% consensus motifs: ‘G-x-LG-xx-P’ (aas 239–245) and ‘Q-xxxxx-G-xxxxx-GQ’ (aas 383–399), the latter also being one of the blocks reported by Mahillon *et al*. [49] and part of the only region in the MPs (aas 364–382) that consistently forms short *α*-helices in AL3 models of different tymovirus MPs. A ‘WW domain’ [50, 51], which binds with Pro-rich proteins, was found in the core of all 107 tymovirus Mtases (residues 218 and 239). Models of multiple copies of MP aa sequences, or of single MPs together with other individual tymovirus proteins, or with the 5′- or 3′-terminal regions of the TYMV genome, showed no increase in ipTM, indicating that they are unlikely to interact or form multimers.

### 115 kDa – the Mtase, IDP2 and Pep protein

AL3 models revealed that the catalytic core of the Mtase enzyme is formed by *α*-helices and *β*-sheets encoded by the N-terminal 330–380 aas; however, it has an additional region of 200–230 aas attached to the 3′ end of the core, which, in turn, is attached to IDP2 and Pep (Fig. 3c–e). Parts of the additional 200–230 aas region have been called the ‘Iceberg region’ by Ahola and Karlin [52], or a broader region, including the Pep, called the ‘Salyut region’ of the okra mosaic virus genome by Stephan *et al*. [53]. This region includes the stretch of 41 aa residues identified by Moriceau *et al*. [54] to be ‘responsible for targeting the viral replication complex to the chloroplast envelope’. The region has a distinctive shape (Fig. 3e), namely, a ‘handle’ of paired distal *β*-sheets and proximal *α*-helices attached to a loop that we call IDP1. The N-terminal *α*-helix of the handle is bent and is the 41 aa residues identified by Moriceau *et al*. [54]. The handle region is largely hydrophobic, whereas the loop is strongly hydrophilic (mean hydrophobicity ~ −0.51). The total length of the structured handle (~8.6–10.2 nm, based on canonical helical and *β*-strand dimensions) is comparable to the thickness of plasma membranes of plant cells (~5–10 nm), consistent with a potential membrane-spanning or membrane-anchoring function. The complete structure is present in TYMV and other tymoviruses and also in related tymovirids (Table S1, available in the online Supplementary Material). These include bombyx mori latent virus which has been cultured in silkworm cells [55] that do not contain chloroplasts; therefore, we distinguish the ‘membrane-targeting domain’ (MTD) from the Tmase to which it is attached.

We used the ‘ipTM score’ of AL3 models to assess whether the 115 kDa protein or parts of it might form polymers (see Methods). There is however a limit of 5,000 aa residues that can be modelled in a single AL3 experiment, and this sets a maximum for each experiment to six copies of the 115 kDa protein (Tmase+IDP2+Pep) but permits larger numbers of its component proteins to be modelled together (Fig. 4).

**Fig. 4.**
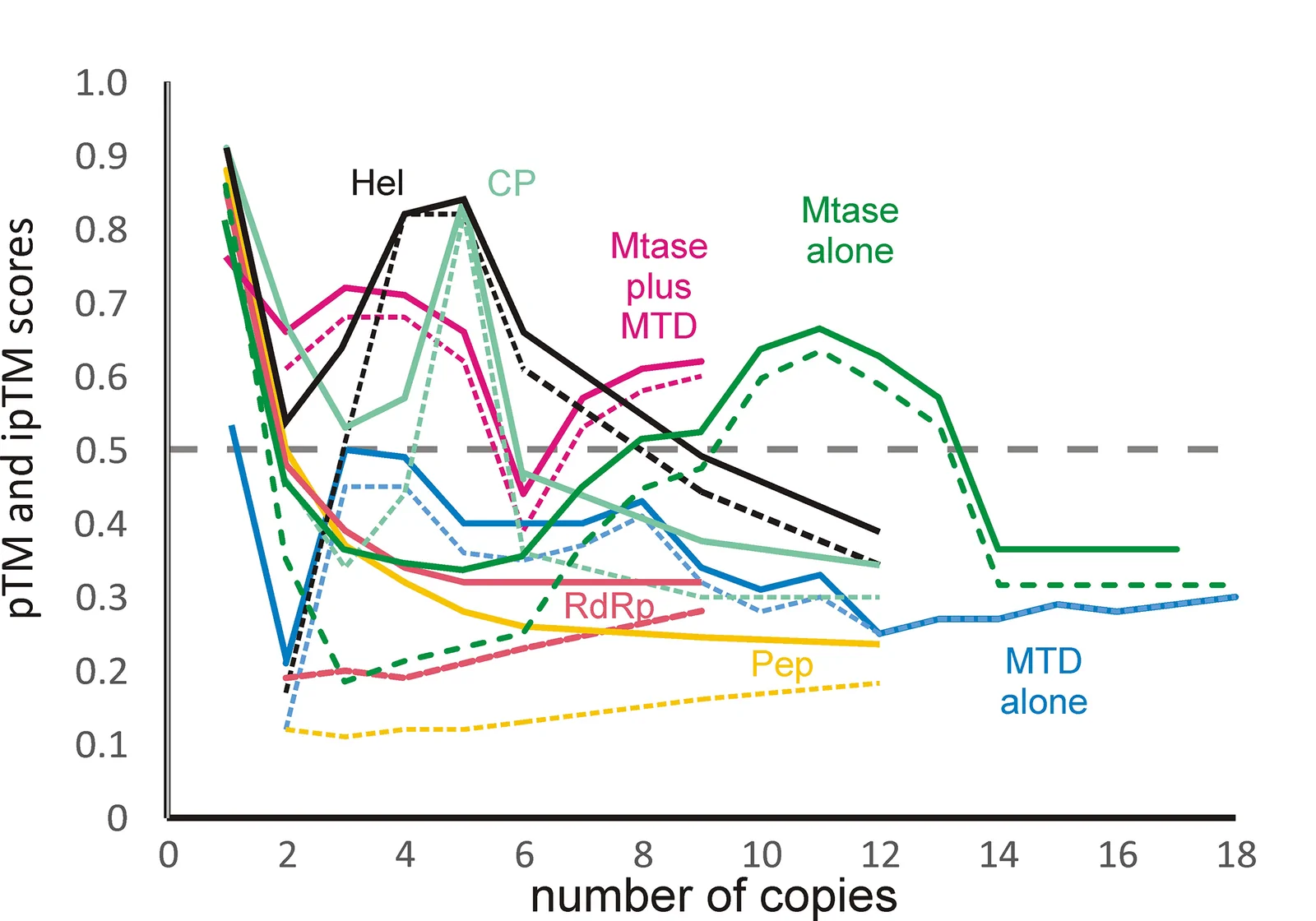
The ‘pTM score’ (unbroken lines) and ‘ipTM score’ (broken lines) (Y-axis) of the models obtained from different numbers of copies of monomers (X-axis). The results for each protein are coloured individually. Peak scores above 0.5 indicate the number of copies of each protein that may form the most stable polymers.

Maximal ipTM and pTM values were obtained when three copies of the Mtase:MTD proteins were modelled together and also when nine or more were modelled together (Fig. 4; red lines). However, when multiple copies of only the Mtase proteins were modelled, the undecamer (11-mer) produced maximal ipTM and pTM values (Fig. 4; green lines), and when multiple copies of only the MTD protein were modelled together, the trimer had maximal ipTM and pTM values (Fig. 4; blue lines). These maxima were associated with differences in the structures of the polymers; when three to five copies of the Mtase:MTD protein were modelled together, they aligned in parallel to form a curved sheet (Fig. 5a, b), whereas six or more copies formed cylinders of Mtases crowned by a fan of MTDs (Fig. 5d, e). These results suggest that as the Mtase:MTD polymerize, the trimeric and undecameric polymers may have specific but different functions.

**Fig. 5.**
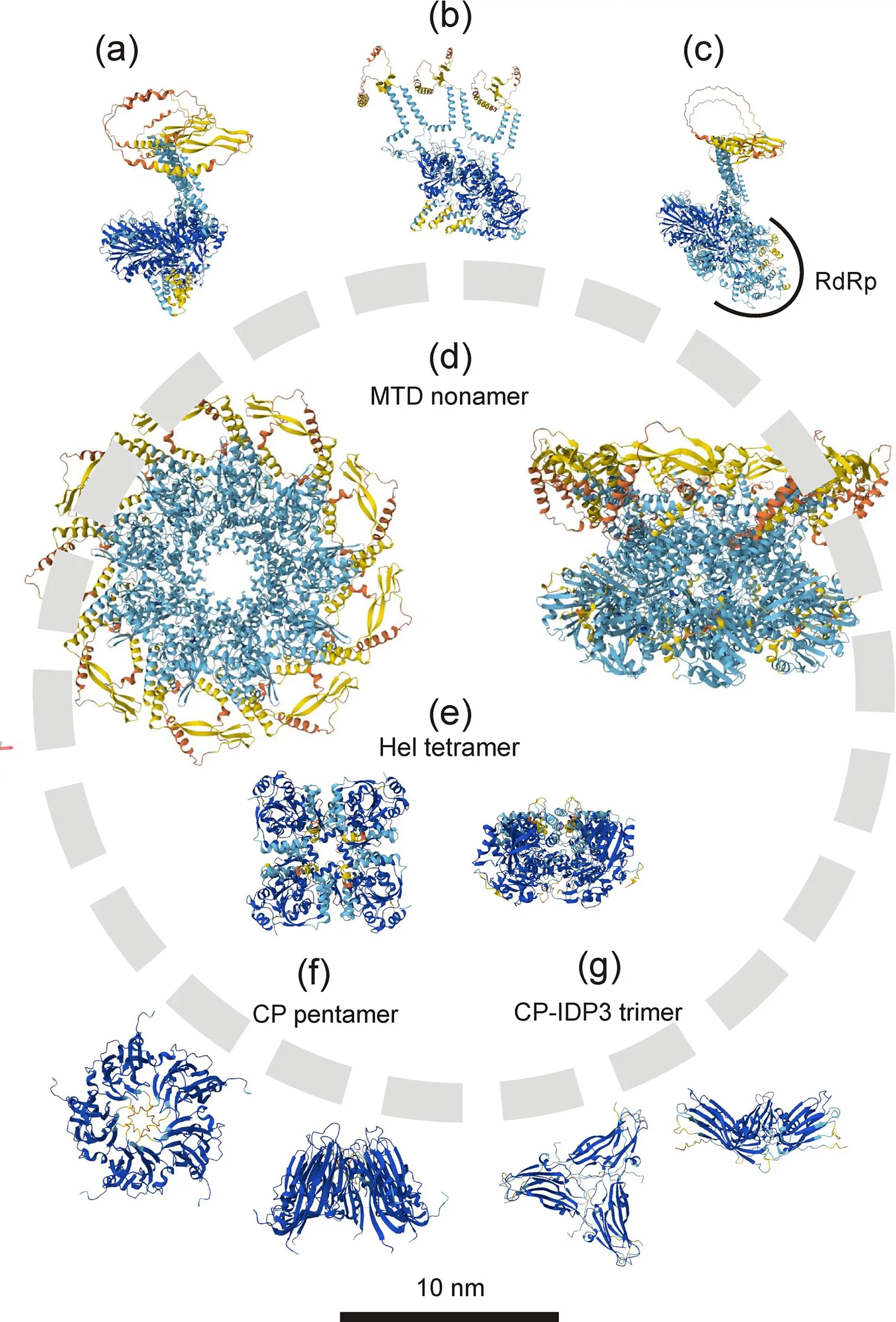
AL3 ribbon diagrams of TYMV proteins. Well-supported structures are in blue, less certain ones in yellow and uncertain in brown. All models are about the same magnification (size bar 10 nm). The grey dashed circle is 28 nm in diameter, matching a tymovirus virion for scale.

Figure 5 shows two views of trimeric Mtase:MTD proteins (Figure 5**a, b**), and a trimer Mtase:MTD modelled together with a single RdRp (Figure 5**c**). It also shows nonamers of the Mtase:MTD protein (Figure 5d).

The Mtase:MTD polymers of six or more monomers resemble grommets; namely, each is ‘a ring of metal or material protecting the edge of a hole through which something such as a thread or rope is put’ (Cambridge Dictionary). The largest computable Mtase:MTD model, a nonamer, was measured using Mol*Viewer [56]. The mouth of the polymer at the MTD end is wider, ca. 17 nm, than the other mouth, ca. 13.5 nm. The groove at the waist of the polymer has a diameter of 9.1 nm. The axis of the Mtase:MTD polymer is ca. 13.2 nm long, and the minimum diameter of the axial hole is ca. 2.9 nm and is at the MTase end of the cylinder. The corresponding radial measurements of undecamers will, of course, be ca. 20% greater. Thus, each Tmase:MTD polymer is approximately the same size as the neck of a tymovirus RO found by Matthews [9–12] (Fig. 1a). The trimeric Mtase:MTDs could be intermediates that carry other proteins to the forming RO. Modelling experiments of three copies of the Mtase:MTD sequences together with single copies of the other tymovirus proteins found that adding a single RdRp molecule (Fig. 5c) produced the best oligomer model (ipTM 0.69, pTM 0.72). In this oligomer, the RdRp nestled into the face of the trimer that forms the lumen of the trimer. Models combining a single copy of the Hel (ipTM 0.62, pTM 0.67) similarly suggested that it too may associate with the Mtase:MTD trimer. However, models combining single copies of the Pep (ipTM 0.55, pTM 0.62) or CP (ipTM 0.38, pTM 0.49) were less well supported, and adding the associated IDPs to the RdRp and CP decreased the scores of them all (see Discussion).

The Mtases and MTDs of APMMV, APLV, DMV, KYMV and OYMV all formed cylindrical polymers like TYMV’s (Fig. S1) with maximal ipTM and pTM values ranging from 0.64 to 0.74 and 0.66 to 0.76, respectively, when they were modelled from 11 monomers.

### 42 kDa protein – the Hel

In AL3 polymer modelling experiments, the Hel protein was found to have the greatest ipTM as a tetramer (Fig. 5e). Each of the tetramers is ca. 10 nm at its widest and ca. 7.25 nm when measured corner to adjacent corner. Its axis is ca. 5.8 nm long. The Hels are a large and diverse group of proteins, and the TYMV Hel is from superfamily 1 that has the so-called Walker A signature of ‘GxxxxGKT’ (residues 91–98) and Walker B signature of ‘ILVIDE’ (residues 153–158) [57]. There are a few reports of such Hels being dimers, but this is probably the first evidence of a superfamily 1 Hel that is tetrameric.

### 66 kDa protein – the RdRp

There was no model evidence that the RdRp formed polymers. The structured RdRp molecule is about 6 nm at its longest dimension. The N-terminus of the RdRp is attached to IDP3, which is 48 aas long and is lipophilic (Fig. 6)

**Fig. 6.**
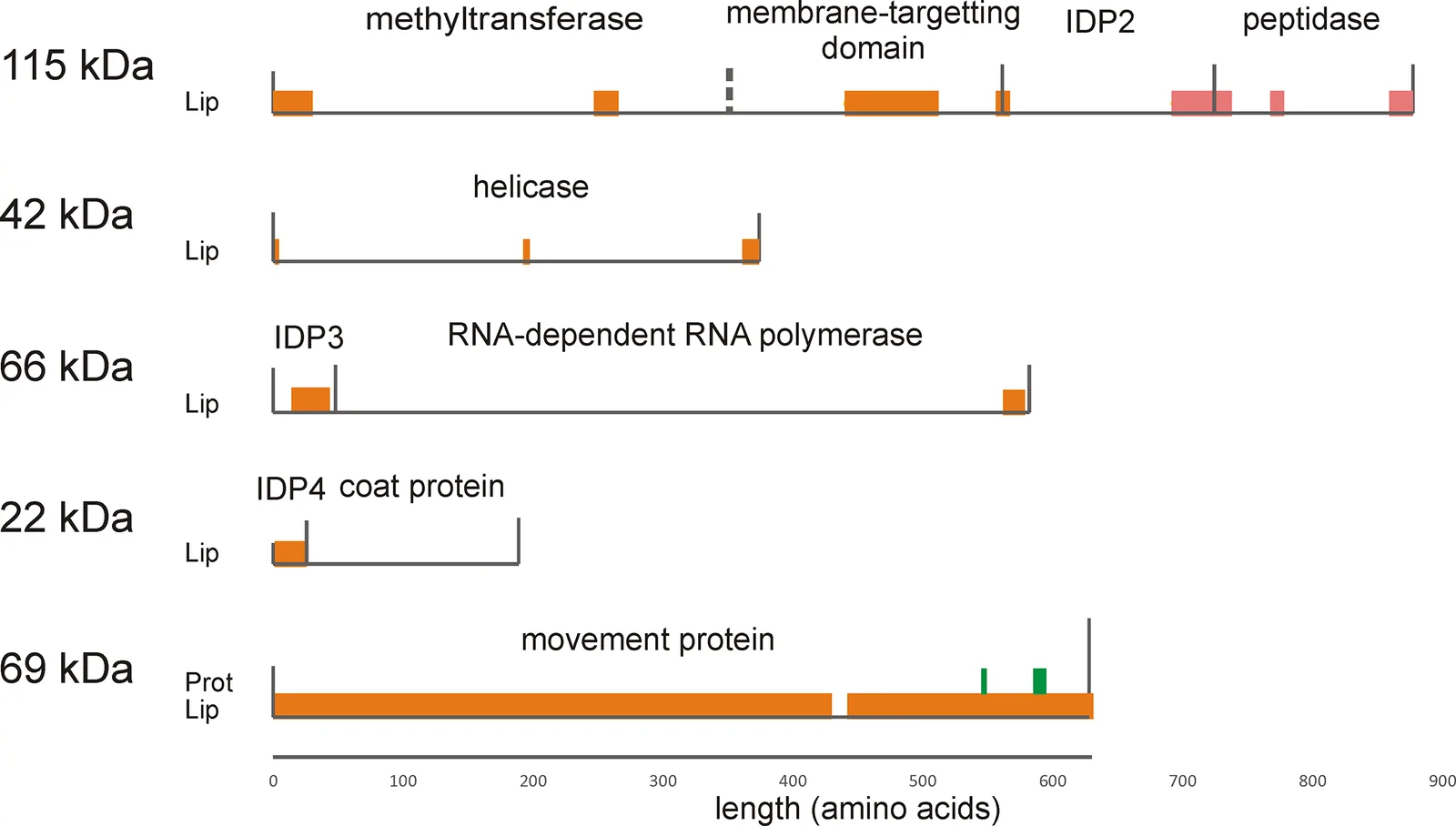
Maps of the proteomes of seven tymoviruses (APLV, APMMV, DMV, ELV, KYMV, OYMV and TYMV) showing the regions likely to interact, on average, with proteins (green) and/or lipids (orange), as predicted by DisoRDPbind and DisoLipPred. Continuous bars show regions where interaction is likely significant and continuous in four or more of the seven different tymoviruses.

### 22 kDa protein – the CP and IDP4

Pentamers of the CP monomer were favoured (Fig. 5f), which are ca. 8.8 nm wide. However, IDP4 is attached to the N-terminus of the CP in all tymovirus virions, and when multiple copies of the IDP4:CP monomers were modelled, they formed trimers (Fig. 5g) with the greatest ipTM support (two copies ipTM 0.42, three 0.68, four 0.49 and five 0.36). Thus, trimers are most likely to be the CP intermediate involved in the assembly of virions.

### Intrinsically disordered proteins

Short regions of helix were present in models of some IDPs, but not all. When multiple copies of the TYMV IDPs, including its MP, were modelled together, all gave unstructured models with pTMs and ipTMs values less than 0.5, indicating that they do not form multimers.

### Ligand binding

We used the tools available at https://biomine2.cs.vcu.edu/, to predict the ligand binding of the proteins APLV, APMMV, DMV, ELV, KYMV, OYMV and TYMV (Fig. 6). These tools have been trained on databases of proteins of eukaryotes and viruses with known binding behaviour and predict the likely binding of proteins, especially IDPs, to classes of ligands, such as nucleic acids, proteins or lipids [58, 59].

The MPs, the largest of the IDPs, had the most complex pattern of binding propensities; their N-terminal two-thirds were lipophilic, and all were predicted to have both protein and nucleic acid-binding regions in the 3′-terminal one quarter of their length, although these predictions were judged to be significant in only five of the viruses (APLV, APMMV, DMoV, ELV and TYMV). DisoLipPred [45] gave the most consistent predictions, especially for binding to lipids, and prediction of binding to proteins and nucleic acids were obtained using DisoRDPbind [46].

The predicted lipid-binding residues of the Mtase–MTD were found to line the upper and lower surfaces of the groove around the waist of the polymer (Fig. 7). These lipophilic regions probably stabilize the polymer around the necks of the ROs in chloroplasts’ outer plasma membrane. Both the Hel and the IDP3–RdRp proteins have N- and C-terminal lipophilic regions. In the Hel tetramer, the lipophilic regions come to lie at the tips of the structure and also in the central hole. In the IDP3–RdRp structure, the lipophilic residues come be adjacent (Fig. 7) and could, for example, confine the RdRp to the inner plasma membrane of the RO. In contrast, the Pep has lipophilic residues distributed broadly across its surface, and the lipophilic residues of the Hel (42 kDa protein) are at the tips of the tetramer (Fig. 7c, d), and both the Hel and the Tmase:MTD polymer contain small lipophilic patches within the constricted ‘throat’ regions of their respective polymers

**Fig. 7.**
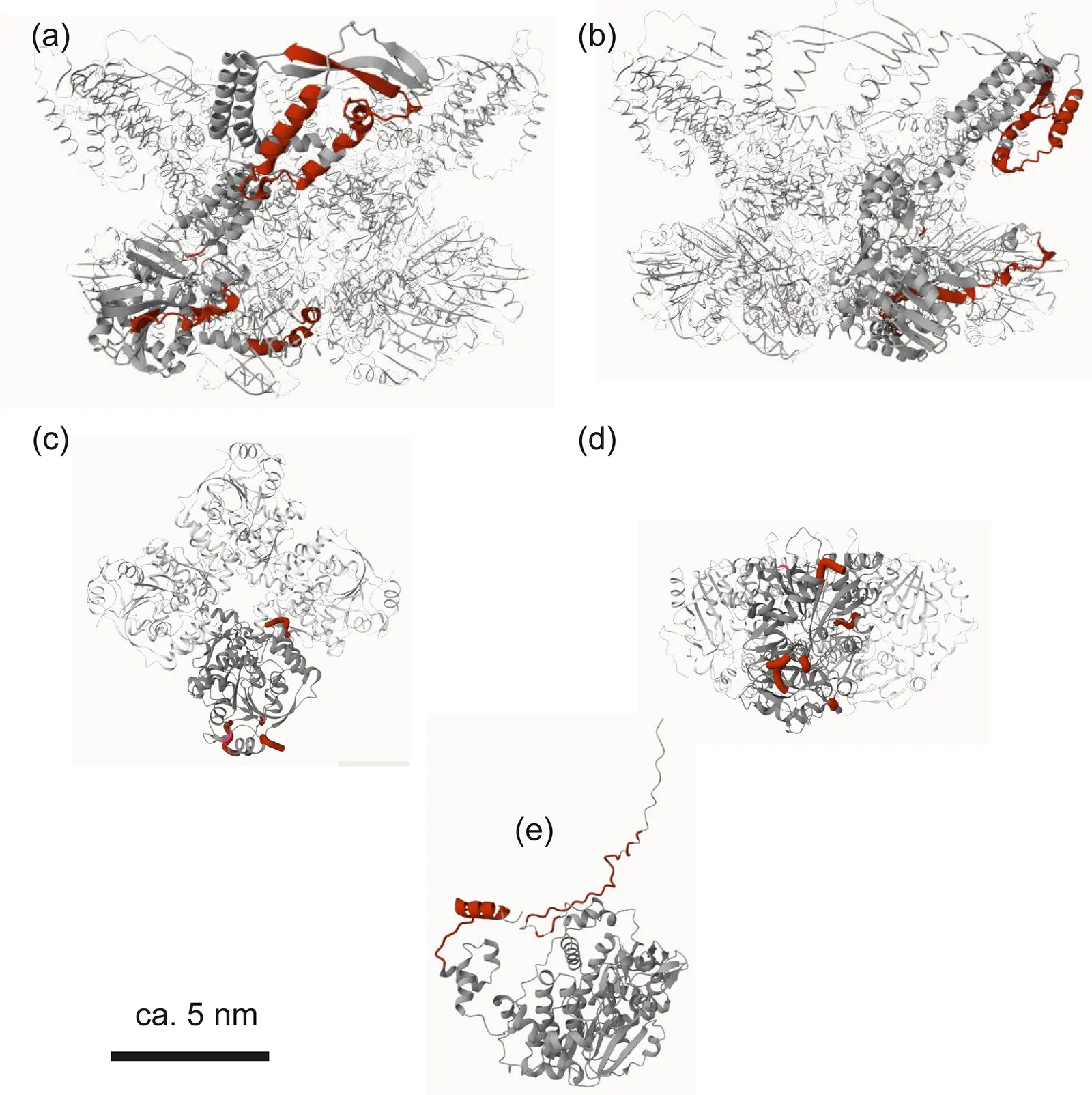
Ribbon diagrams of TYMV Mtase:MTD multimeric proteins and Hel, with the lipophilic residues of a single protein coloured red. Also IDP3:RdRp showing the unstructured IDP3 region extending from the folded RdRp domain.

The IDP4 region of the 22 kDa CP precursor (IDP4–CP) is unexpectedly lipophilic, and this supports the possibility that virion assembly may involve the chloroplast outer membrane, as suggested by Hatta and Matthews [10].

## Discussion

Early micrographic and biochemical studies of TYMV showed that infected cells have rounded and clumped chloroplasts with small vesicles on the inner surfaces of their outer membranes (Fig. 1a–c) [8–10, 60]. Several lines of biochemical evidence have shown that the vesicles are the site of replication of the virus. Prod’Homme *et al*. [61], for example, showed that the RdRp is located in the chloroplast periphery and functions only when the Mtase:MTD:IDP2:Pep polypeptides (i.e. 115 kDa protein), together with Hel, are present. Furthermore, Garnier *et al*. [62] showed immunocytochemically that the RdRp and the Mtase:MTD:IDP2:Pep protein were in both the outer envelope of infected chloroplasts and in the cytoplasm. Thus, these vesicles may be the tymovirus version of the ROs found in other positive-sense ssRNA viruses.

The Mtase:MTD polymers, we describe, probably form the necks of the vesicles (Fig. 1a–c). The ring of material around each neck (Fig. 1d) and the main body of the polymer are ca. 15 nm in diameter, and the apparently uniform size of the vesicles may be linked to the structurally preferred number of 11 Mtase:MTDs forming each polymer. However, when complete and functioning as ROs, molecules and their precursors would have to enter and leave each vesicle through the 3.5 nm wide axial hole in its polymer neck. This would not hinder a ssRNA that had been unwound by a Hel, nor a completely base-paired dsRNA, as these have ‘hydrated effective widths’ of 1.0–1.2 and 2.5–2.6 nm, respectively. However, this suggests that Hel and RdRp molecules are too large to pass through the polymer neck and so can only enter a vesicle while it is forming. Thus, our finding that sheets of three Mtase:MTD molecules significantly bind a single RdRp molecule (ipTM 0.69, pTM 0.72; Fig. 5) or Hel (ipTM 0.62, pTM 0.67; Fig. 5) suggests that they might enter ROs while they are forming by binding to trimeric Mtase:MTD molecules.

Finally, one must consider whether there is enough space in each vesicle for the molecules it may contain. The vesicles, as seen in sections, seem to be no more than ca. 80 nm in diameter, and this is confirmed by the freeze-fractured surfaces of infected chloroplasts (Fig. 1c) which show the vesicle necks in quasi-hexagonal arrays with the closest ca. 75 nm apart. The internal space of each vesicle (Fig. 1b) can be seen to have a diameter of ca. 60 nm and hence a volume of at least 10^5^ nm^3^. The Hel and RdRp are each only ca. 100 nm^3^/molecule (ca. 5 nm in diameter), and although negative genomes and progeny genomes of 6,400 nts have a volume of ca. 1.9×10^3^ nm^3^, their effective hydrated volume could be one hundred times greater, depending also on how base-paired they are [63, 64]. Thus, the vesicle could accommodate multiple copies of the viral proteins, but it may contain only a few negative-strand copies of the genome, perhaps only one; however, recombinant tymoviruses have been found [25].

Hatta and Matthews [60] described a zone ca. 60 nm thick of electron-lucent material (Fig. 1e) between the outer membrane of infected chloroplasts and densely packed virions in the plasmolemma. The tissue they examined had been plasmolysed with sucrose before fixation, so its thickness before treatment is uncertain. However, in our model, this is the region where the long and flexible ‘proteophilic’ IDP2s, each with a terminal Pep protein, would hang from the Tmase:MTD polymers, possibly protecting molecules exiting the area of the RO against the host’s ubiquitin proteasome defenses [65, 66]; IDP2s are from 44 to 209 aa long (Table 1) and, hence, 16–75 (mean 61) nm long.

Plasmodesmata are likely to be the other major site of viral activity in tymovirus-infected plant tissue. These link adjacent cells and are involved in local spread of a virus within an infected plant [67, 68]. Bransom [21] showed that although TYMV virions are required for systemic spread throughout an infected plant, the CP is not required for cell-to-cell spread, suggesting that the virus spreads locally as genomes rather than virions. Furthermore, the MP is required for spread between cells, but is not required for replication in protoplasts [22]. We note that tymoviruses spread to adjacent cells without having caused visible changes to the plasmodesmata as none of the micrographic studies of tymovirus-infected plants report changes to the plasmodesmata. Thus, tymovirus MPs may assist cell-to-cell spread merely by associating lengthwise with genomic RNAs, when both are fully extended while moving through plasmodesmata. MPs are the most lipophilic of TYMV’s proteins, and their binding propensities are more complex than those of the other TYMV proteins as their C-terminal region binds proteins and has a short nucleic acid-binding region (Fig. 6). Plasmodesmata are 30–50 nm in diameter and usually at least 100 nm long [68]. They are lined by a thin cytoplasmic sleeve and have a central desmotubule, ca. 15 nm in diameter, that is part of the endoplasmic reticulum of the cells they link. The desmotubule and the wall plasma membrane are separated by a fluid cytoplasmic sleeve, which although divided into nano-channels, only 2–3 nm wide, would probably allow a ssRNA and IDP chain to pass, as a ssRNA molecule, that is not base-paired, is 1.0–1.2 nm wide, and an IDP chain has a diameter of 0.5–0.7 nm, giving a combined width of 1.5–1.9 nm. The MPs of different tymoviruses range from 576 to 758 codons (200–300 nm) in length, and their genomes are 6,400 nts (3.6 µm) long. Therefore, a fully extended genome would require 13–18 MPs to protect it when moving from cell to cell, and this might also explain how post-translational gene silencing of TYMV is evaded in *Arabidopsis* [23], because short dsRNAs are required to activate the Dicer system [69].

In summary, we have shown that AL3 modelling, augmented by ‘binding propensity’ tests, yields useful predictions of the likely structure of the tymovirus proteins in the virocell. They focus on events at a scale between those obtained from biochemical experiments and those from microscopy. We have used them to attempt to connect observations on tymoviruses, especially TYMV, obtained in biochemical studies and electron microscopy, and they have provided a useful window on possible events in the ROs and plasmodesmata of tymovirus-infected plants.

## Supplementary material

10.1099/jgv.0.002329 Supplementary Material 1.

## Abbreviations

AL3: AlphaFold3
APLV: Andean potato latent virus
APMMV: Andean potato mild mottle virus
CP: coat protein
DMoV: dulcamara mottle virus
ELV-Cz: erysimum latent virus
Hel: helicase
IDP: intrinsically disordered protein
ipTM: interface predicted template modelling
KYMV: kennedya yellow mosaic virus
MP: movement protein
Mtase: methyltransferase
MTD: membrane-targeting domain
OYMV: ononis yellow mosaic virus
Pep: peptidase
pTM: predicted template modelling
RdRp: RNA-dependent RNA polymerase
RO: replication organelles
TYMV: turnip yellow mosaic virus
