# Supplementary material for: An in silico study of tymovirus virocells
Source: J Gen Virol. 2026 Oct 1;107(10):002329. doi: 10.1099/jgv.0.002329 (PMC13630270; doi:10.1099/jgv.0.002329)
Supplement: Supplementary file 1 [file jgv-107-02329-s001.pdf]

Supplementary Material Fig 1

AL3 models of polymers of the Mtase:MTD proteins of selected tymoviruses – acronyms as in text. The polymers are viewed from the side of each cylindrical polymer; MTDs above Tmases. Colours: blue structures are strongly supported (Mtases), less certain yellow and regions of uncertain structure are brown. The number of copies of the Mtase:MTD protein in each model were limited by data restriction of AL3, however the likely optimum ipTM would be obtained with 11 or 12 copies

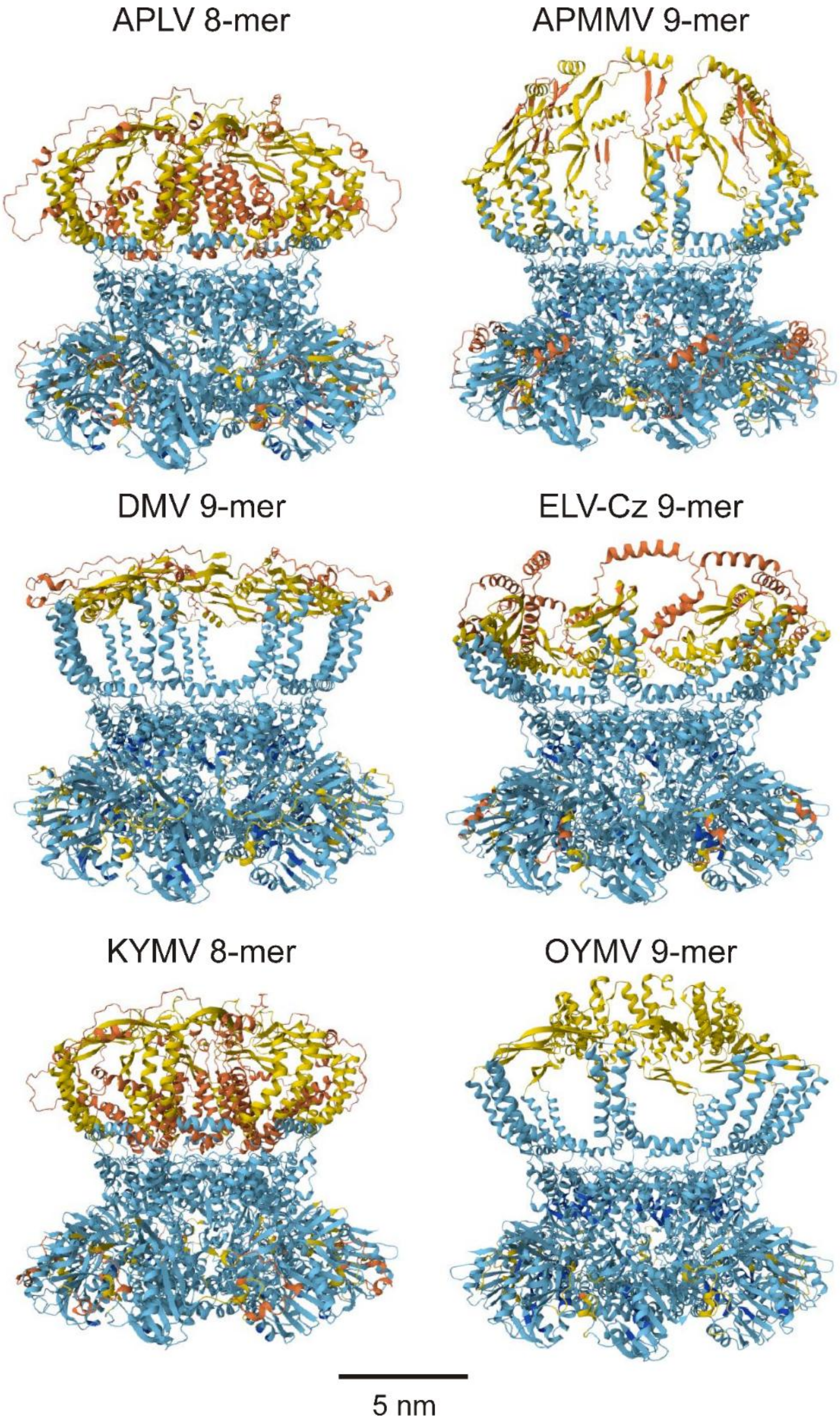

Supplementary Material Table 1. A list of the viral Mtases which an AlphaFold3 model showed to have the 'membrane targeting domain' (MTD) found in TYMV, and others that did not.

Tymoviruses				
	Andean potato latent		NC_020470	
	Dulcamara mottle		NC_007609	
	Erysimum latent		PP093825	
	Kennedy yellow mosaic		NC_001746	
	Turnip yellow mosaic		NC_004063	
	Inya insect-associated virus-1		MW251315	
Maculavirus				
	Grapevine fleck		NC_003347	
Marafiviruses				
	Maize rayado fino		NC_002786	
	Oat blue dwarf		NC_001793	
Tymovirus-like				
	Bombyx mori latent		NC_038331	
	Bombyx mori macula-like		NC_015524	
	Culex-originated Tymoviridae-like		JQ429443	
	Ek Balam		MH822863	
	Myotis brandtii tymo-like		OP880199	
	Poinsettia mosaic		NC_002163	
Alphaflexiviruses				
	Allexivirus	Alfalfa virus S	NC_034622	MTD not present
	Potexvirus	Potato virus X	NC_007289	MTD not present
Betaflexivirus				
	Capillovirus	Apple stem grooving	NC_001749	MTD not present
	Carlavirus	Potato virus S	NC_007289	MTD not present
	Foveavirus	Apple stem pitting	NC_003462	MTD not present
	Trichovirus	Apple chlorotic leaf spot	M58152	MTD not present
